# Stromal integrin α11-deficiency reduces interstitial fluid pressure and perturbs collagen structure in triple-negative breast xenograft tumors

**DOI:** 10.1186/s12885-019-5449-z

**Published:** 2019-03-15

**Authors:** Hilde Ytre-Hauge Smeland, Ning Lu, Tine V. Karlsen, Gerd Salvesen, Rolf K. Reed, Linda Stuhr

**Affiliations:** 1 Department of Biomedicine, University of Bergen, P.O. Box 7804, 5020 Bergen, Norway; 2 Centre of Cancer Biomarkers, Norwegian Centre of Excellence, University of Bergen, P.O. Box 7804, 5020 Bergen, Norway

**Keywords:** Integrin α11β1, Interstitial fluid pressure, Cancer associated fibroblasts, Collagen organization, Triple-negative breast cancer

## Abstract

**Background:**

Cancer progression is influenced by a pro-tumorigenic microenvironment. The aberrant tumor stroma with increased collagen deposition, contractile fibroblasts and dysfunctional vessels has a major impact on the interstitial fluid pressure (PIF) in most solid tumors. An increased tumor PIF is a barrier to the transport of interstitial fluid into and within the tumor. Therefore, understanding the mechanisms that regulate pressure homeostasis can lead to new insight into breast tumor progression, invasion and response to therapy. The collagen binding integrin α11β1 is upregulated during myofibroblast differentiation and expressed on fibroblasts in the tumor stroma. As a collagen organizer and a probable link between contractile fibroblasts and the complex collagen network in tumors, integrin α11β1 could be a potential regulator of tumor PIF.

**Methods:**

We investigated the effect of stromal integrin α11-deficiency on pressure homeostasis, collagen organization and tumor growth using orthotopic and ectopic triple-negative breast cancer xenografts (MDA-MB-231 and MDA-MB-468) in wild type and integrin α11-deficient mice. PIF was measured by the wick-in-needle technique, collagen by Picrosirius Red staining and electron microscopy, and uptake of radioactively labeled 5FU by microdialysis. Further, PIF in heterospheroids composed of MDA-MB-231 cells and wild type or integrin α11-deficient fibroblasts was measured by micropuncture.

**Results:**

Stromal integrin α11-deficiency decreased PIF in both the orthotopic breast cancer models. A concomitant perturbed collagen structure was seen, with fewer aligned and thinner fibrils. Integrin α11-deficiency also impeded MDA-MB-231 breast tumor growth, but no effect was observed on drug uptake. No effects were seen in the ectopic model. By investigating the isolated effect of integrin α11-positive fibroblasts on MDA-MB-231 cells in vitro, we provide evidence that PIF regulation was mediated by integrin α11-positive fibroblasts.

**Conclusion:**

We hereby show the importance of integrin α11β1 in pressure homeostasis in triple-negative breast tumors, indicating a new role for integrin α11β1 in the tumor microenvironment. Our data suggest that integrin α11β1 has a pro-tumorigenic effect on triple-negative breast cancer growth in vivo. The significance of the local microenvironment is shown by the different effects of integrin α11β1 in the orthotopic and ectopic models, underlining the importance of choosing an appropriate preclinical model.

**Electronic supplementary material:**

The online version of this article (10.1186/s12885-019-5449-z) contains supplementary material, which is available to authorized users.

## Background

Triple-negative breast cancer (TNBC) is defined by the absence of estrogen receptors, progesterone receptors and HER-2 amplification and represents an aggressive breast cancer subtype. Despite significant advancements in the treatment of other breast cancer subtypes, there is still no licensed targeted therapy available for the treatment of TNBC, and therefore little improvement in survival has been observed for this patient population over the last years [1, 2]. This highlights the need for better understanding of TNBC and identification of mechanisms involved in disease progression and treatment response.

It is now well recognized that breast cancer progression can be influenced by a pro-tumorigenic microenvironment surrounding the malignant epithelial cells. This environment consists of a heterogeneous mixture of stromal cells, including a diversity of cancer associated fibroblasts (CAFs), a biological active network comprising the extracellular matrix (ECM), in addition to the interstitial fluid and its solutes [3, 4]. New knowledge about the components of the microenvironment and how they interact with tumor cells can hopefully identify new biomarkers or potential targets in TNBC.

The aberrant stroma affects the physiological forces within the tumor. Indeed, the hydrostatic pressure in the tumor interstitium, known as interstitial fluid pressure (PIF), is considerably increased in the majority of solid tumors [5], including human breast cancer [6, 7], and this poses a major physiological barrier to transport of soluble factors within the tumor [8].

Increased PIF has been shown to predict poor prognosis in some solid tumors [9, 10], and can also hinder effective delivery of drugs into the tumor [11–13]. Finding ways to lower tumor PIF may therefore increase efficiency of cancer therapy.

Fibroblasts can actively modify PIF and transcapillary fluid exchange (reviewed in [8, 14, 15]) and the molecular mechanisms are outlined by collagen contraction assays [16, 17] and heterospheroids [18–20], as well as parallel in vivo experiments [21–23]. Dysfunctional blood and lymph vessels will lead to fluid accumulation in the tumor interstitium, and swelling of hyaluronan and proteoglycans would in normal conditions hinder an increase in PIF [8, 24]. Tension exerted by fibroblasts and collagen network can probably counteract this swelling, resulting in a persistent increased PIF [14]. However, although fibroblast-mediated contraction has previous been shown to be dependent on β1-integrins [21], fibroblast-mediated PIF influence is still not fully understood.

Integrin α11β1 is a collagen binding integrin expressed during differentiation of myofibroblasts [25–27] and is involved in collagen organization [17, 28] and tumor stiffness [28]. As a collagen organizer and a link between contractile fibroblasts and the complex collagen network, integrin α11β1 could be a regulator of tumor PIF. Although a few studies indicate that it has a physiological role in the regulation of PIF in dermis [29, 30], its influence on PIF in tumors remains to be demonstrated. A better understanding of the mechanisms that regulate pressure homeostasis within a tumor, can probably lead to a new insight into breast carcinogenesis, and we therefore investigated the effect of stromal integrin α11-deficiency on pressure homeostasis, ECM organization and tumor growth using two human TNBC xenograft models.

## Methods

### Cell lines

MDA-MB-231 (ATCC® HTB-26™) was provided by Professor James Lorens (University of Bergen, Bergen, Norway), and MDA-MB-468 (ATCC® HTB-132™) was obtained from the American Type Culture Collection (Manassas, VA., USA). The MDA-MB-231 cells were fingerprinted before use and matched with the cell line MDA-MB-231 (ATCC® HTB-26™) in the ATCC database. MDA-MB-231 was used at passage number five to nine, while the MDA-MB-468 cells were used at passage number two to five. These TNBC cell lines have high tumor take in SCID mice and slowly forming tumors, which may be more stromal dependent than more rapidly growing xenografts. Wild type (WT) and integrin α11-deficient (α11-KO) mouse embryonic fibroblasts (MEFs) were obtained from mouse embryos of embryonic day 14.5 as described previously [31]. In order to obtain immortalized MEFs, primary MEF cultures were infected with recombinant retrovirus-transducing simian virus 40 (SV40) [32]. All cell lines were grown in Nutrient Mixture F-12 Ham (Sigma-Aldrich, Steinheim, Germany) supplemented with 10% Foetal Bovine Serum, 100 units/ml penicillin, 100 μg/ml streptomycin, and 1–2% L-glutamine (all from Sigma-Aldrich). The cells were grown as single monolayers in a humidified incubator at 37 °C in 5% CO_2_ and in all experiments used at log phase. All cell lines tested negative for mycoplasma contamination.

### Xenograft models

The integrin α11-deficient heterozygous SCID mouse strain was generated as previously described [28]. PCR-genotyping was performed on DNA extracted from ear biopsies [32]. The animals were kept in individually ventilated cages, cared for regularly and efforts were made to age- and weight match the animals. All animal experiments were approved by the Norwegian Food Safety Authority (Permit Number 20168751) which is the competent body responsible for authorizing research projects in animals in Norway. This is in accordance with the EU directive 2010/63 article 36.

A total of 5 × 10^5^ MDA-MB-231 or 1.5 × 10^5^ MDA-MB-468 tumor cells in 0.15 ml PBS were injected into the fourth mammary fat pad (orthotopic), and for the MDA-MB-231 also subcutaneously on the mouse flank (ectopic). Tumor size was measured using a caliper. The tumor volume was calculated using the formula*; tumor volume (mm*^*3*^*)* = *(π/6) × a*^*2*^ *× b,* where *a* represents the shortest diameter and *b* represents the longest diameter of the tumor. All animals were anesthetized using Isofluran (Isoba®vet. 100%, Schering-Plough A/S, Farum, Denmark) and eventually sacrificed by cervical dislocation under deep anesthesia. For investigation of the primary tumor, all the MDA-MB-231 injected mice were sacrificed day 57 post injection. For the MDA-MB-468 injected mice, some of the tumors showed tendency to ulcerate the skin, and these mice were sacrificed immediately. To make the groups comparable, one mouse from the opposite group and with similar tumor load was sacrificed on the same day.

To evaluate metastatic spread to the lungs, they were processed and fixed as previously described [33] (*n* = 5 WT and 5 α11-KO and *n* = 5 WT and 4 α11-KO for the MDA-MB-231 and MDA-MB-468 injected mice, respectively).

All measurements and analysis in this study were performed blinded to genotype.

### Measurement of interstitial fluid pressure

The wick-in-needle technique was used to measure the tumor PIF [34]. Briefly, a standard 23-gauge needle with a side hole filled with nylon floss and saline was connected to a PE-50 catheter, a pressure transducer and a computer for pressure registrations, using the software Powerlab chart (version 5, PowerLab/ssp. AD instruments, Dunedin, New Zealand). The needle was inserted into the central part of the tumor after calibration. After a period of stable pressure measurements, the fluid communication was tested by clamping the catheter which shall cause a transient rise and then return to pressure prior to clamping. Measurements were accepted if the pre- to post-clamping value was within ±1 mmHg.

PIF in heterospheroids was measured with the micropuncture technique described previously [18]. Briefly, the spheroids were collected and transferred to 10-cm Lysine-coated cell culture dishes (Nunc, Thermo Fisher, Waltham, MA., USA) and left to attach for 2 h at 37 °C. PIF was measured using sharpened glass capillaries (tip diameter 3–5 μm) connected to a servo-controlled counter pressure system. The glass capillaries were filled with hypertonic saline (0.5 M) colored with Evans blue dye and inserted into the central parts of the spheroid with the help of a stereomicroscope (Wild M5, Heerbrugg, Switzerland). PIF in the cell culture medium directly outside the spheroid was defined as the zero reference pressure.

### Electron microscopy of collagen fibrils in the tumor

Tumor samples were taken from the tumor periphery and were fixed and processed as previously described [33]. A JEM-1230 Transmission Electron Microscope (TEM) (Jeol, Tokyo, Japan) was used to measure the diameter and organization of the collagen fibrils, and images from four to six different areas of the tissue were analyzed. Pictures were captured at × 100,000 magnification and analyzed using Image J 1.46 (National Institute of Health, Bethesda, MD., USA) to measure the fibril diameter. To investigate the organization of the collagen fibrils, pictures were captured at × 30,000 magnification and scored from one to four considering collagen fibril organization and alignment within the collagen fibers.

A JSM-7400F Scanning Electron microscope (Jeol) was used to study the tumor collagen fibril scaffold architecture. Five images from different areas of the tumor were captured from each tumor at × 10,000 magnification.

### Immunostaining and Picrosirius-red staining

Histological analysis was performed on both paraffin embedded sections and cryosections. For paraffin embedded sections, 5 μm thick sections were deparaffinizated and rehydrated, followed by heat induced antigen retrieval at pH 6 (#S1699, Dako, Agilent, Santa Clara, CA., USA) for Ki67 (100 °C, 20 min) and αSMA (100 °C, 25 min), pH 9 (#2367, Dako) for Coll III (100 °C, 25 min) or pH 10 (#T6455, Sigma Aldrich) for PDGFRβ (110 °C, 5 min). After antigen retrieval, the sections were incubated with peroxidase block (#K006, Dako) and then primary antibody. Envision+ System-HRP (#K4006 or #K4010, Dako) was used as secondary antibody, in addition to rabbit anti-goat for collagen III (1:1000, #6164–01, Southern Biotech, Birmingham, AL., USA), and DAB was used as chromogen, except for αSMA staining, where a FITC-conjugated antibody was used. Analysis of immunohistochemistry was performed using Leica DN 2000 Led (Leica Microsystems, Wetzlar, Germany). The following primary antibodies were used on paraffin sections: rabbit anti-mouse PDGFRβ mAb (1:100, #3169, Cell Signaling Technology, Danvers, MA., USA), goat anti-mouse Type III Collagen pAb (1:100, #1330–08, Southern Biotech), anti-mouse αSMA mAb (F3777, dilution 1:200, Sigma Aldrich) and mouse anti-human Ki67 mAb (1:100, #M7240, Dako).

Cryosections with a thickness of 6 μm were fixed in ice-cold methanol (− 20 °C, 8 min) and rehydrated with PBS, followed by blocking with 10% goat serum. Afterwards, the following primary antibodies were supplied: rabbit anti-mouse integrin α11 pAb (1:200, custom-made, Innovagen AB, Lund, Sweden, [31]), mouse anti-human cytokerain AE1/AE3 mAb (1:200, #M3515, Dako) and mouse anti αSMA mAb (1:200, #A5228, Sigma Aldrich). Goat anti-rabbit Alexa 594 (1:400, #111–585-144, Jackson ImmunoResearch, Ink., West Grove, PA., USA) and goat anti-mouse Alexa 488 (1:400, #315–545-045, Jackson ImmunoResearch) were used as secondary antibodies. Mounting was done with ProLong Gold Antifade Mountant with DAPI (#P36934, ThermoFisher). The staining results were evaluated under an Axioscope fluorescence microscope and micrographs were acquired using a digital AxioCam MRm camera (Zeiss, Oberkochen, Germany).

Picrosirius-red stain (Polysciences inc, Warrington, FL., USA) was used for a semi-quantitative measurement of collagen type I and III as previously described [33].

### Evaluation of the staining

For Picrosirius-red, collagen III, PDGFRβ and αSMA, a total of four to six pictures were captured from each tumor with × 100 magnification. Images were taken in the tumor periphery in order to avoid the necrotic central area. The software Image J 1.46 (National Institute of Health, Bethesda, MD., USA) was used to identify the amount of positive pixels.

For Ki67, the tumors were examined using light microscopy with an eye-piece grid at × 630 magnification. A total of 500 tumor cells from the tumor periphery were evaluated, and distinct nuclear staining regardless of intensity was registered as positive. Areas with necrosis, bleeding or inflammation were avoided.

### Microdialysis

Microdialysis was performed as previously described [35] on the MDA-MB-231 mammary fat pad tumors. Briefly, after anesthesia with Ketalar (Pfizer Inc., NY., USA) and Dormitor (Orin Pharma AS, Espoo, Finland), one microdialysis probe was placed in the MDA-MB-231 mammary fat pad tumor (CMA12 Elite Microdialysis probe, ref.nr 8,010,434) and one in the jugular vein (CMA12 Elite Metal free, ref.nr 80,111,204). The probes were connected to a PE-50 catheter, perfused by a pump (CMA100 Microinjection pump, ref.nr 8,210,040) at a rate of 1 μl/min and left to stabilize for 30 min. After intravenous injection of 0.15 ml 0.65 MBq ^3^H-5FU (Nycomed Amersham, Buckinghamshire, UK), dialysate was sampled and pooled every 10 min for a total of 90 min. Scintillation counting solution (Optiphase Hisafe 3, PerkinElmer, Inc., Waltham, MA., USA) was added, and the radioactivity measured using a liquid scintillation analyzer (Tri-Carb 2900TR, PerkinElmer, Inc.). The probes and pump were delivered by CMA Microdialysis AB, Kista, Sweden.

The area under the curve (AUC) for the plasma and tumor was calculated with Graph Pad Prism 7 (GraphPad Software Inc., La Jolla, CA., USA) as the total radioactivity collected, i.e. as the product of radioactivity (counts per minute) and time. Finally, transport of ^3^H-5FU was expressed as AUC tumor divided by AUC plasma.

After each experiment, the probes were tested in saline with a known amount of ^3^H-5FU, and experiments with probes that differed more than 15% in permeability were excluded.

### Heterospheroids

Heterospheroids containing a mixture of SV40-immortalized MEFs and MDA-MB-231 cells were prepared using the hanging drop method as described previously [19]. Briefly, sub-confluent cells were trypsinized and suspended in culture medium to a concentration of 1 × 10^6^/ml. The MEFs (WT or integrin α11-KO MEFs) and MDA-MB-231 cell suspensions were then mixed at a ratio of 4:1 to make WT MEFs + MDA-MB-231 and α11-KO MEFs + MDA-MB-231 spheroids. Approximately 40 drops (25 μl/ drop, 2.5 × 10^4^ cells/drop) were dispensed onto a lid of a cell culture dish. The lid was then inverted and placed over a cell culture dish containing medium for humidity, and cultured in a humidified incubator at 37 °C in 5% CO_2_ for 5 days.

### Statistical analysis

Sigmaplot 13.0 (Systat Software Inc., Chicago, IL., USA) and Graph Pad Prism 7 (GraphPad Software) were used for statistical analysis. Either the unpaired two-tailed t-test or the Mann- Whitney U test, was used to analyze statistical differences between the two groups. Results were accepted as statistically different when *p* < 0.05. Data are given as mean ± SD, and number of measurements (n) refers to number of tumors or heterospheroids unless otherwise specified.

## Results

### Effect of stromal integrin α11β1 on breast tumor growth

**Fig. 1:**
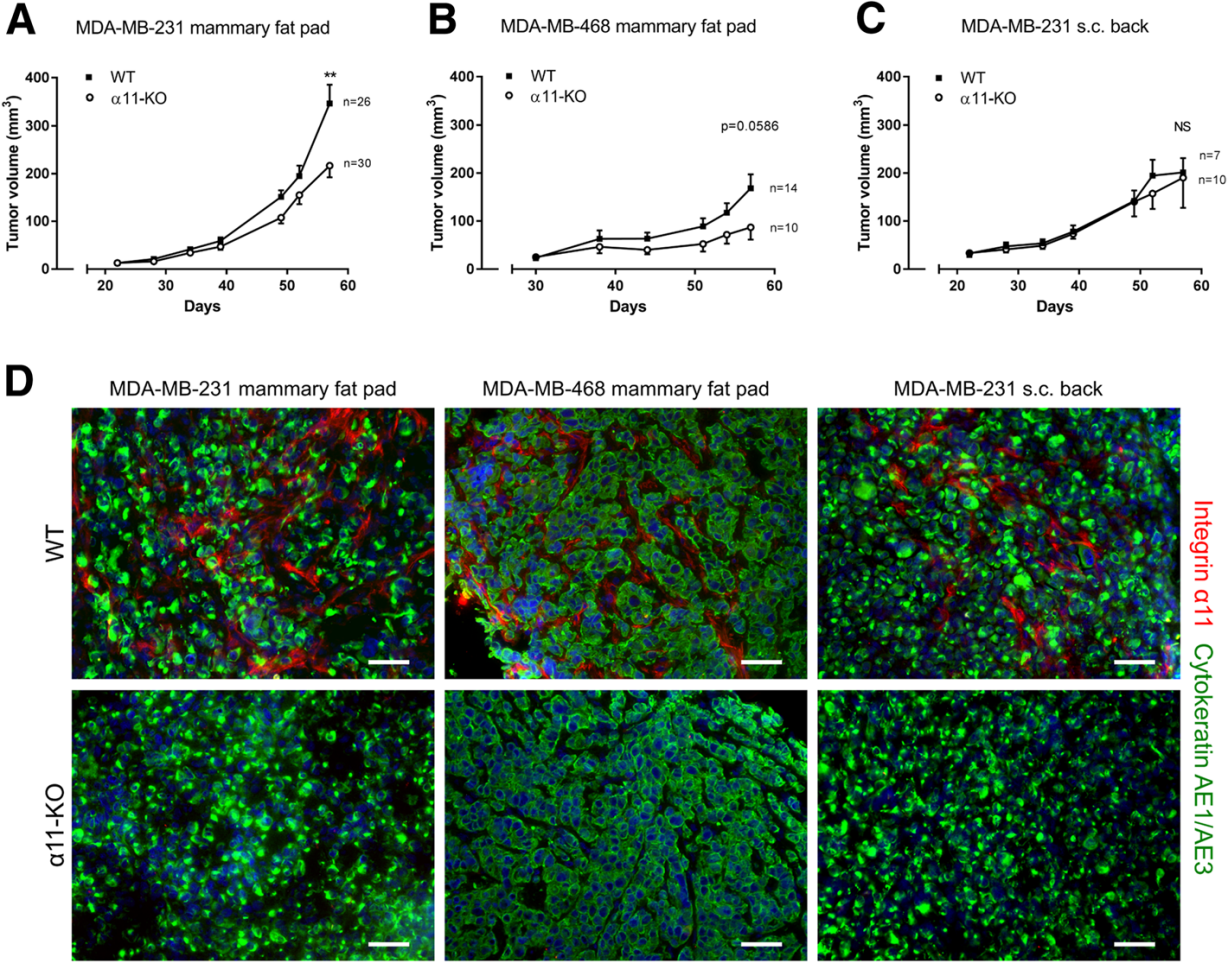
Tumor growth**.** The growth of MDA-MB-231 and MDA-MB-468 xenograft tumors (**a-c**) in WT and α11-KO mice. A total of 5 × 10^5^ MDA-MB-231 and 1.5 × 10^6^ MDA-MB-468 cells were injected into the mammary fat pad, and for MDA-MB-231, also subcutaneously (s.c.) on the back. All MDA-MB-231 injected mice were sacrificed at day 57 post injection. The MDA-MB-468 injected mice were sacrificed at different time points starting with *n* = 20 WT and *n* = 16 α11-KO. Mean ± SEM. ** *p* < 0.01. Immunofluorescence staining of integrin α11 (red), cytokeratin AE1/AE3 (green) and DAPI (blue) in MDA-MB-231 and MDA-MB-468 xenograft tumors (**d**) in WT and α11-KO mice. Scale bars indicate 50 μm

MDA-MB-231 and MDA-MB-468 tumor cells were injected into WT and α11-KO mice. As expected, we found that integrin α11 was expressed in the tumor stroma in WT mice, but not in α11-KO mice (Fig. 1d). Furthermore, the immunofluorescent staining of integrin α11 (Fig. 1d) did not show differences in the amount of integrin α11 expression between the MDA-MB-231 orthotropic and subcutaneous model (*n* = 3–5). The tumor volumes in MDA-MB-231 mammary fat pad tumors were significantly reduced (*p* < 0.01) in α11-KO mice compared to tumors grown in WT mice during their 57 days growth period (Fig. 1a). A clear tendency towards reduced MDA-MB-468 mammary fat pad tumor growth was also seen, but this did not reach statistical significance (*p* = 0.059) (Fig. 1b). Of note, there was no difference in MDA-MB-231 tumor growth when the cells were injected subcutaneously on the back (Fig. 1c).

**Fig. 2:**
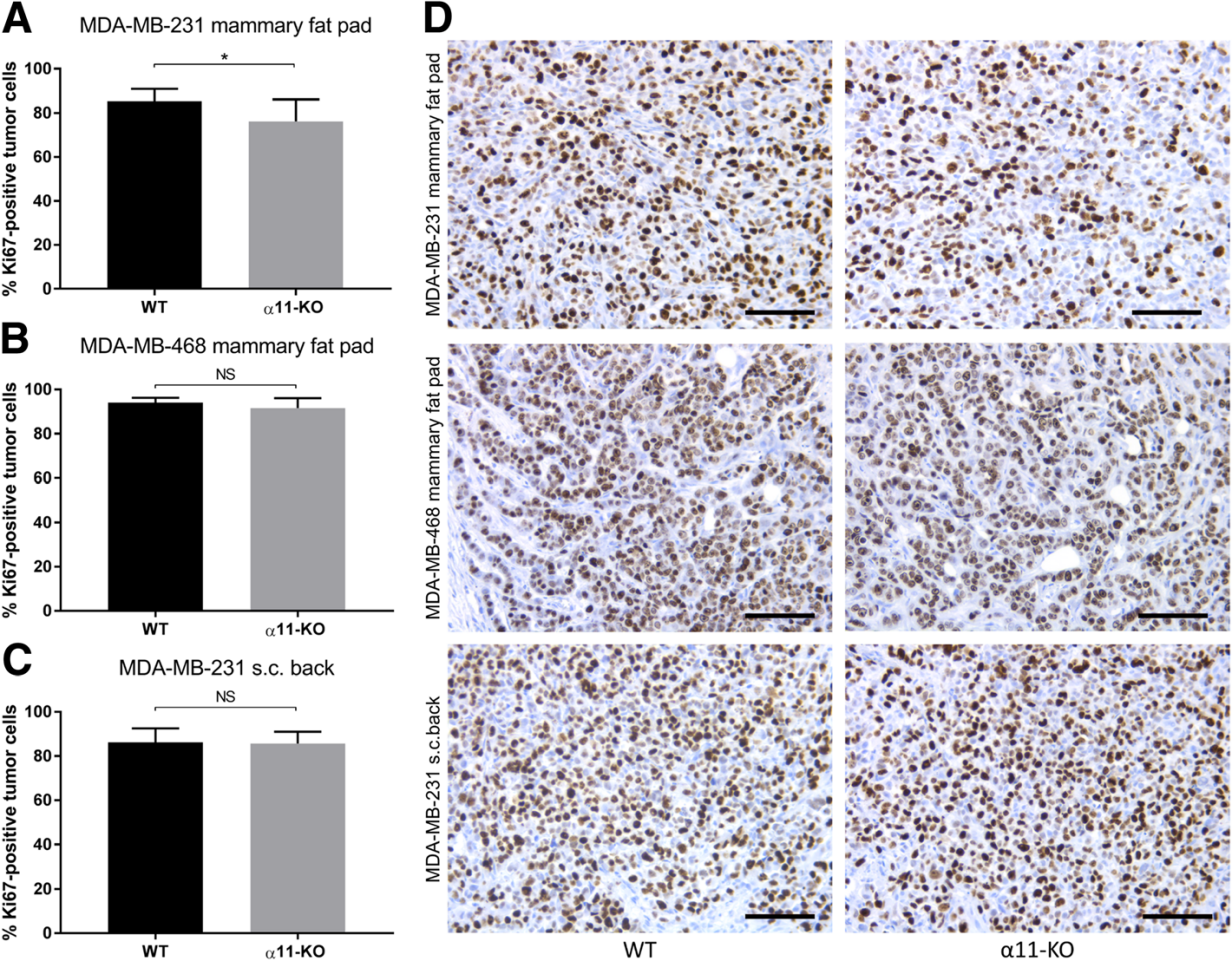
Tumor cell proliferation. The fraction of Ki67-positive tumor cells in MDA-MB-231 and MDA-MB-468 xenograft tumors (**a-c**). Reduced percentage of Ki67-positive tumor cells was only seen in MDA-MB-231 mammary fat pad tumors in α11-KO mice compared to WT (*n* = 7 for MDA-MB-231 and MDA-MB-468 mammary fat pad tumors, and *n* = 4 WT and *n* = 5 α11-KO for MDA-MB-231 subcutaneous tumors). Mean ± SD. * *p* < 0.05. Representative images of Ki67 staining of sections from all xenograft tumors in WT and α11-KO mice (**d**). Scale bars indicate 100 μm

In the MDA-MB-231 mammary fat pad tumors, there was a slight, but statistically significant difference in the number of proliferating tumor cells, indicated by positive Ki67 staining (Fig. 2a and d). However, in the two other tumor models, there were no significant differences in number of proliferating tumor cells (Fig. 2b-d).

### Integrin α11-deficiency reduces tumor interstitial fluid pressure

**Fig. 3:**
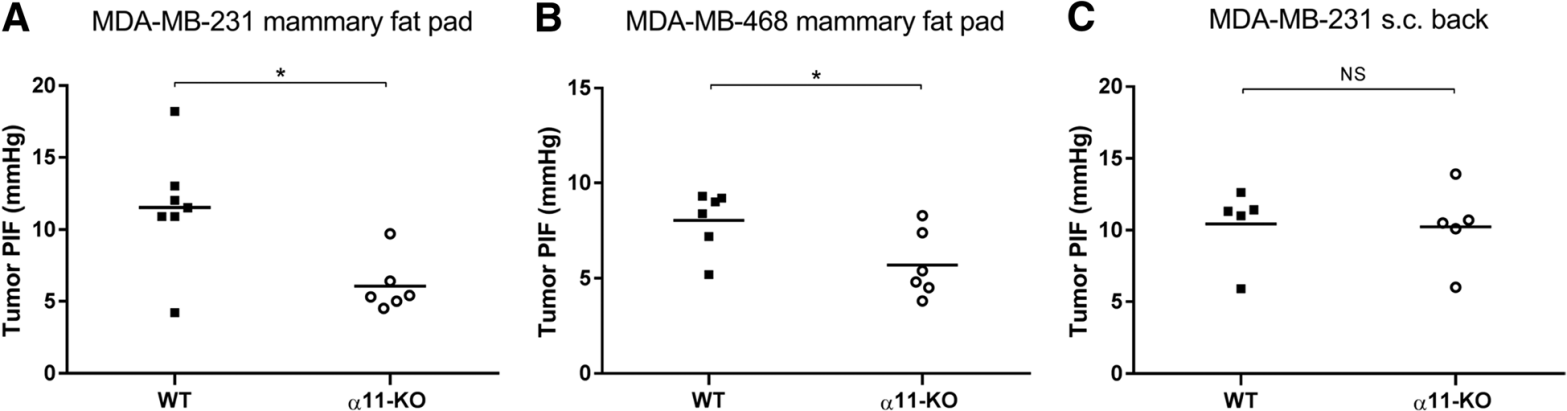
Tumor interstitial fluid pressure. The individual interstitial fluid pressures (PIF) in MDA-MB-231 and MDA-MB-468 xenograft tumors (**a-c**) in WT and α11-KO mice. The horizontal lines indicate the mean values. Reduced tumor PIF was seen in MDA-MB-231 and MDA-MB-468 mammary fat pad tumors in α11-KO mice compared to WT, but no difference was seen in the MDA-MB-231 subcutaneous tumors. * *p* < 0.05

The tumor PIF was measured by the wick-in-needle method. PIF was significantly reduced in both MDA-MB-231 and MDA-MB-468 mammary fat pad tumors grown in α11-KO mice compared to WT (Fig. 3a-b). No difference in PIF was seen in the MDA-MB-231 subcutaneous tumors (Fig. 3c).

### Integrin α11-deficiency perturbs collagen structure

**Fig. 4:**
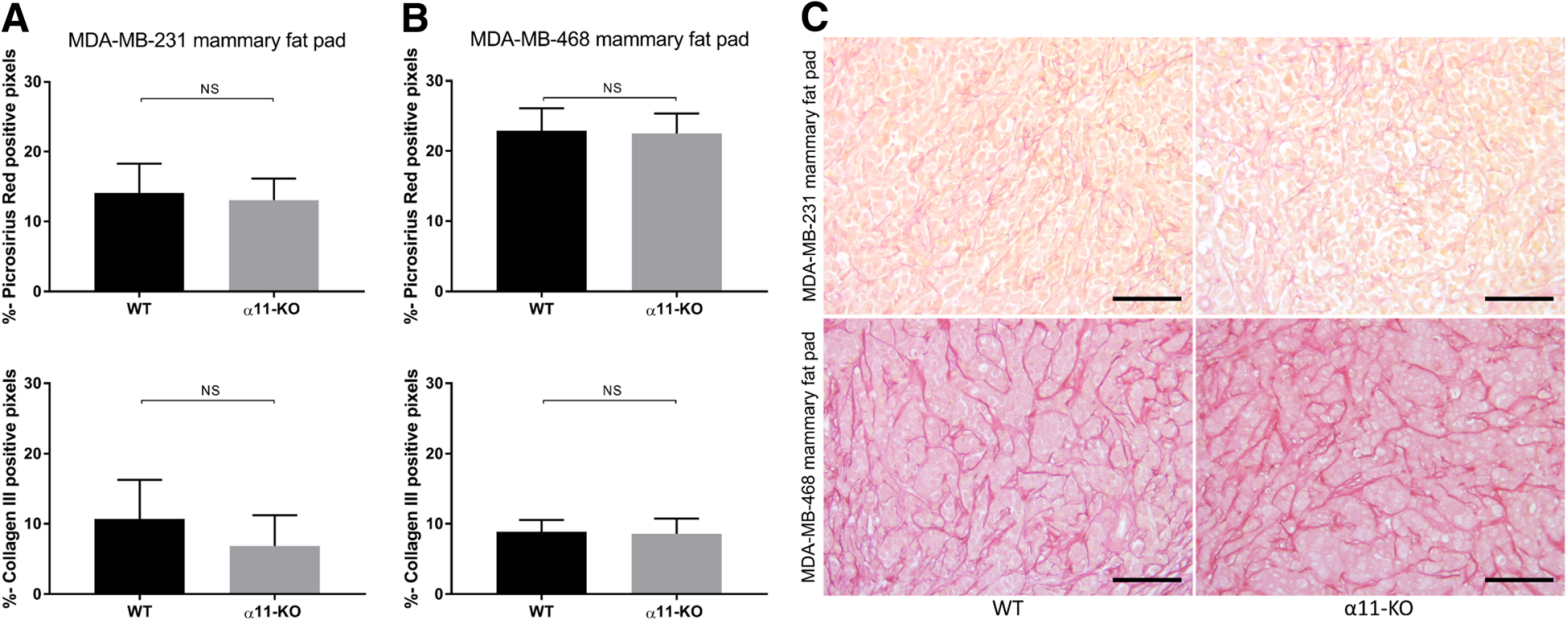
Collagen amount. The total fraction of Picrosirius-red and collagen III positive staining quantified in MDA-MB-231 (**a**) and MDA-MB-468 mammary fat pad tumors (**b**) in WT and α11-KO mice showed no differences (*n* = 6 in both models). Mean ± SD. Representative images of Picrosirius-red staining of sections from MDA-MB-231 and MDA-MB-468 mammary fat pad tumors in WT and α11-KO mice (**c**). Scale bars indicate 100 μm

Picrosirius-red and collagen III staining did not demonstrate differences in the amount of collagen in either of the tumor models (Fig. 4a-c and Additional file 1: Figure S1).

**Fig. 5:**
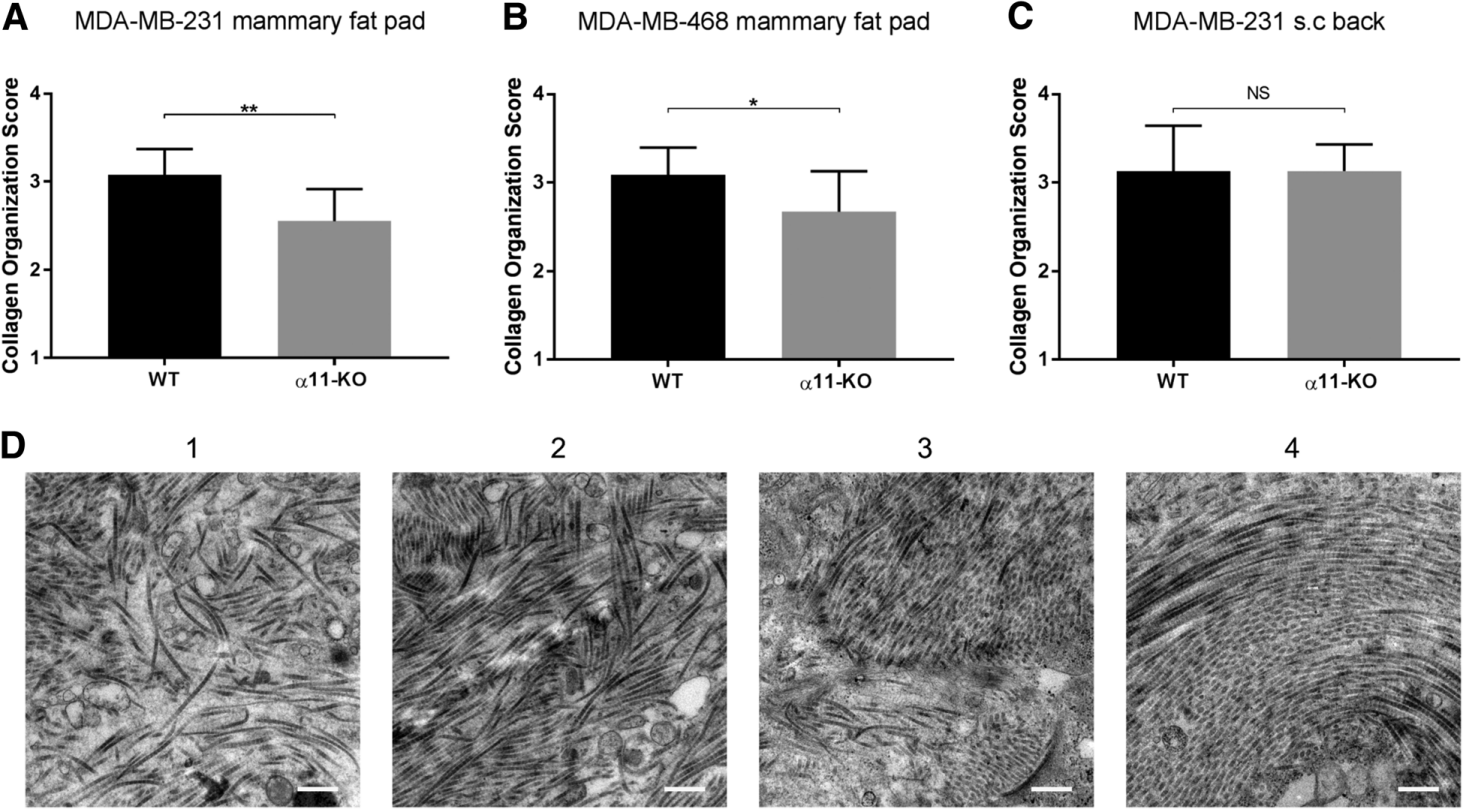
Collagen fibril organization. Transmission electron microscopy pictures from the xenograft tumors were scored from 1 to 4 according to collagen organization. The average collagen organization score per tumor in MDA-MB-231 (**a**) and MDA-MB-468 mammary fat pad tumors (**b**) were lower in α11-KO mice compared to WT (*n* = 8 WT and *n* = 7 α11-KO mice in both models). No difference was found in the MDA-MB-231 subcutaneous tumors (**c**) (*n* = 5). Mean ± SD. * *p* < 0.05, ** *p* < 0.01. Examples of the different scoring values from MDA-MB-468 tumors are shown in (**d**) (1- highly disorganized, 2-moderately disorganized, 3-moderately organized and 4-highly organized). Scale bars indicate 0.5 μm

**Fig. 6:**
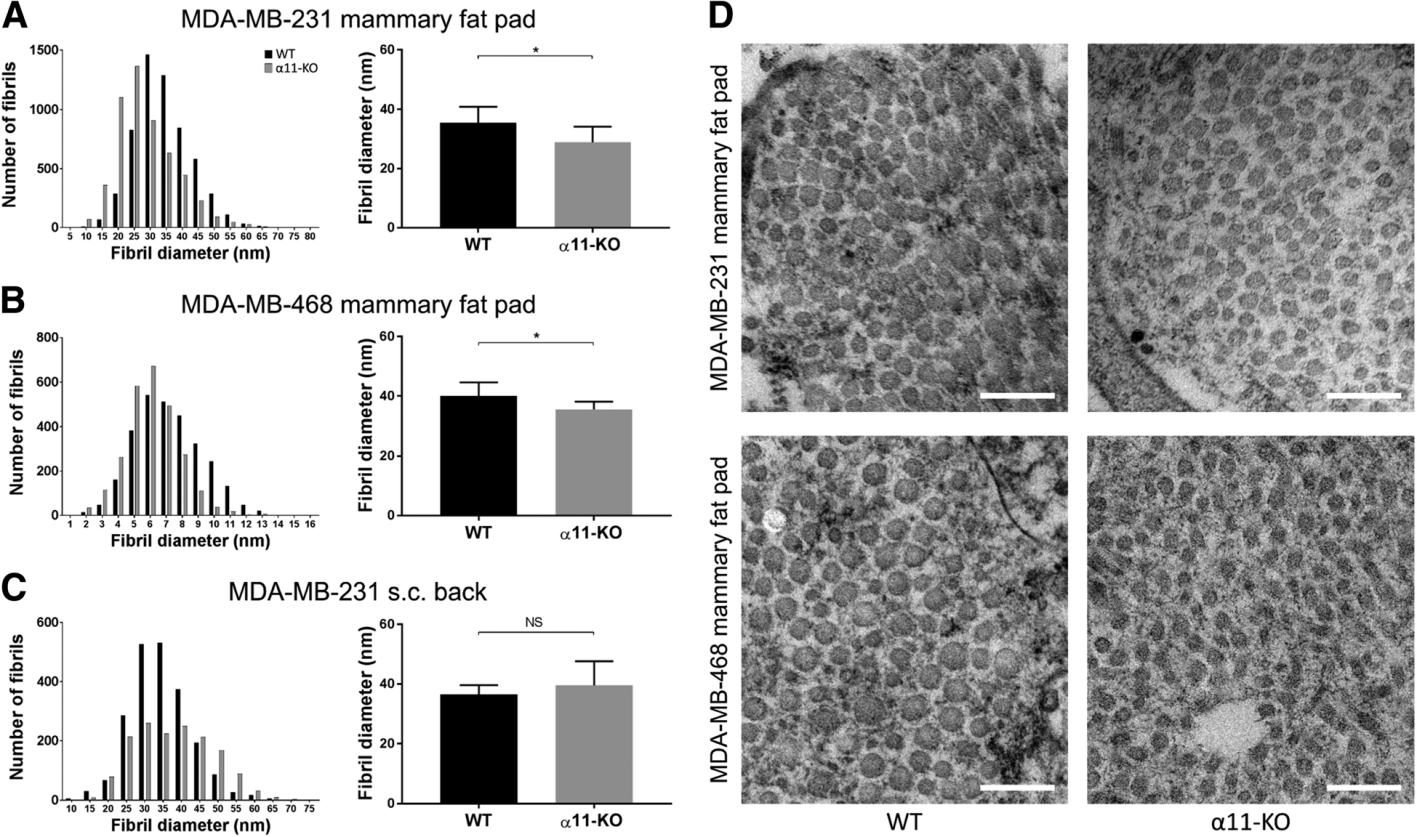
Collagen fibril diameter. Transmission electron microscopy (TEM) was used to analyze collagen fibrils. Collagen fibril diameter distribution and average fibril diameter per tumor in MDA-MB-231 (*n* = 7) (**a**) and MDA-MB-468 mammary fat pad tumors (*n* = 7) (**b**) showed a shift towards thinner fibrils in α11-KO mice compared to WT. No difference was seen in MDA-MB-231 subcutaneous tumors (**c**) (*n* = 5). Mean ± SD. * *p* < 0.05. Representative TEM images of collagen fibrils in MDA-MB-231 and MDA-MB-468 mammary fat pad tumors in both genotypes (**d**). Scale bars indicate 0.2 μm

**Fig. 7:**
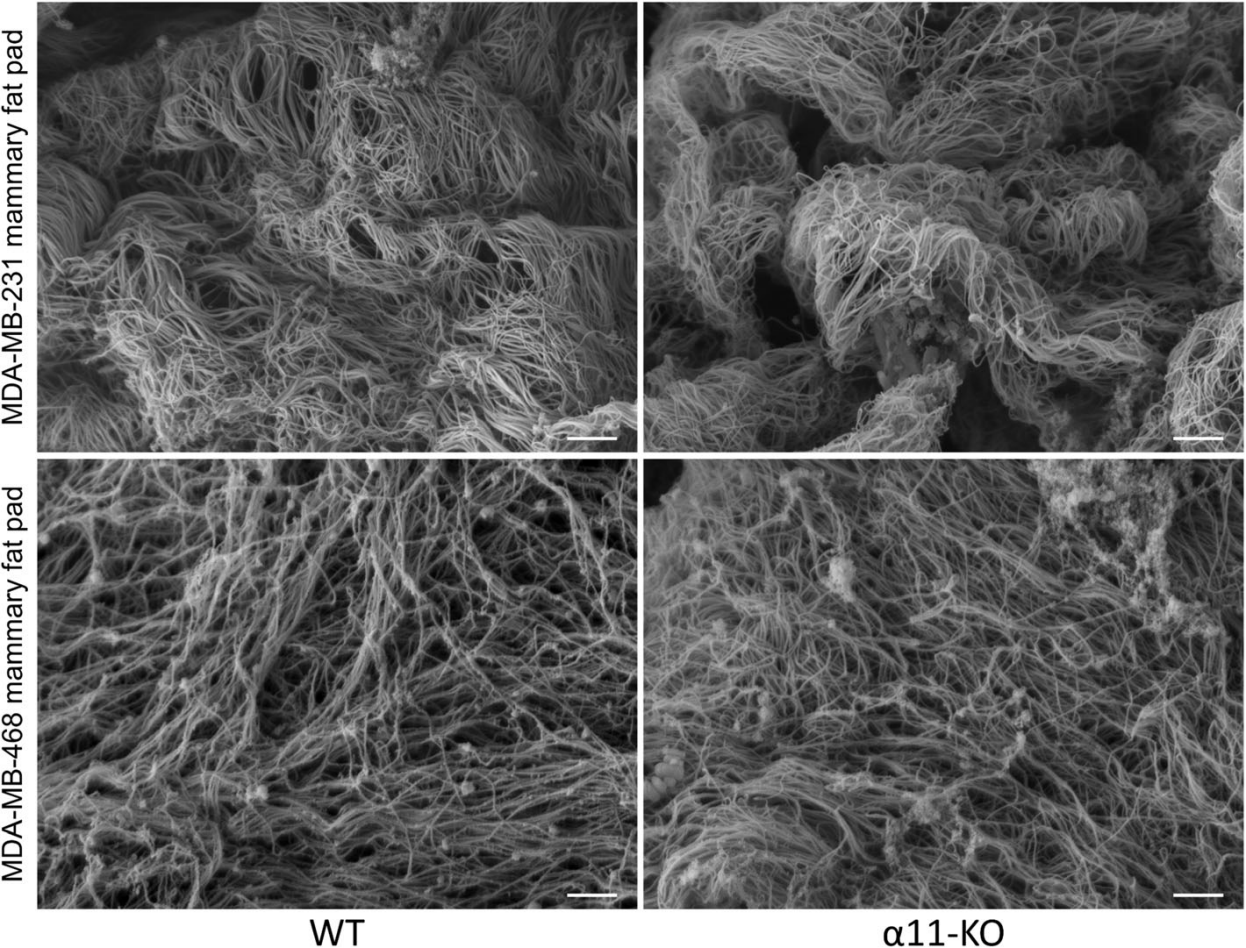
Collagen fibril architecture. Representative scanning electron images of collagen fibrils in MDA-MB-231 and MDA-MB-468 mammary fat pad tumors in WT and α11-KO mice (*n* = 5 WT and *n* = 4 α11-KO in both models). Scale bar indicates 1 μm

Collagen fibril organization and structure in the xenograft tumors were investigated using TEM. As seen in Fig. 5a-b and d, integrin α11-deficiency lead to more disorganized collagen fibril architecture with fewer aligned collagen fibrils in both the MDA-MB-231 and MDA-MB-468 mammary fat pad tumor models. In these tumors, there was also a shift towards thinner collagen fibrils in α11-KO compared to WT mice (Fig. 6a-b and d). No difference was seen in either collagen fibril alignment or collagen fibril diameter in the MDA-MB-231 subcutaneous tumors when comparing α11-KO mice with WT (Figs. 5c and 6c). In addition, SEM did not demonstrate visual differences in the collagen fibril structure between tumors grown in α11-KO mice versus WT (Fig. 7).

**Fig. 8:**
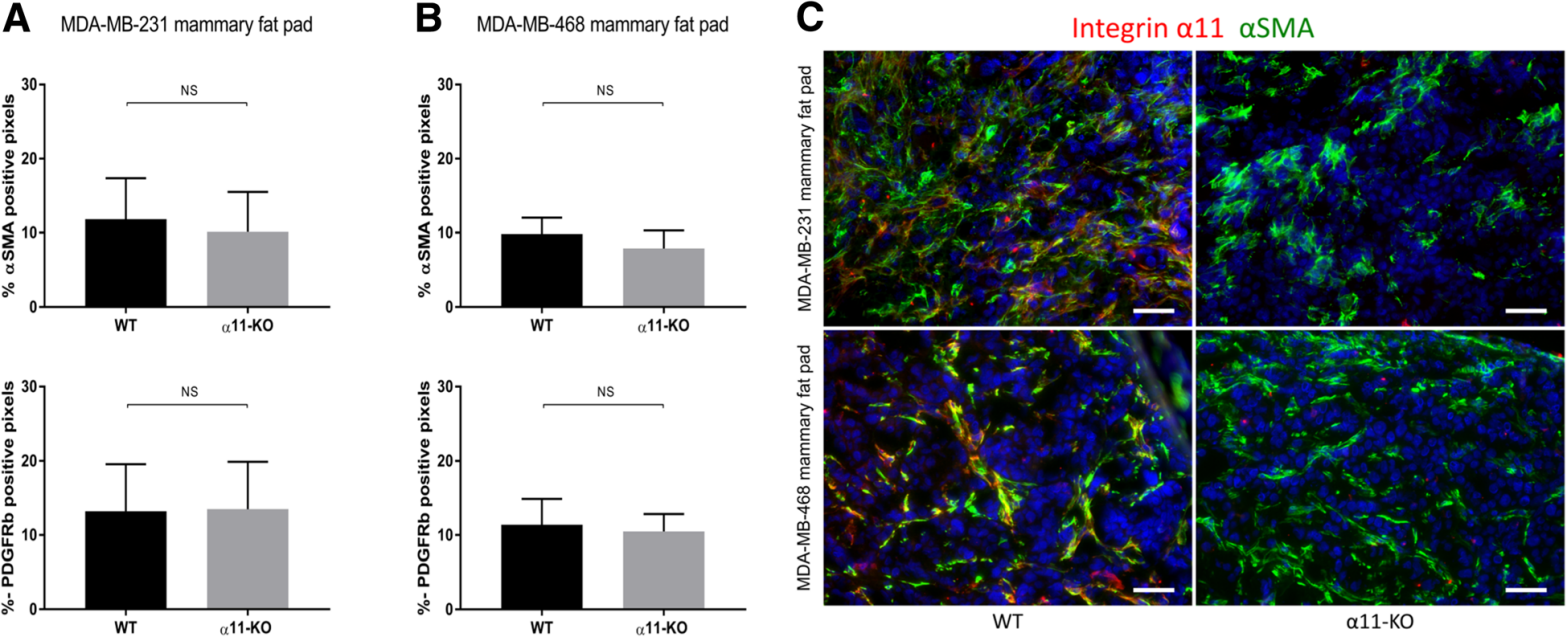
αSMA and PDGFRβ. The total fraction of αSMA and PDGFRβ positive staining quantified in MDA-MB-231 (**a**) and MDA-MB-468 mammary fat pad tumors (**b**) in WT and α11-KO mice showed no differences (*n* = 6 in both models). Mean ± SD. Immunofluorescence staining of integrin α11 (red), αSMA (green) and DAPI (blue) in MDA-MB-231 and MDA-MB-468 mammary fat pad tumors in WT and α11-KO mice (**c**). Scale bars indicate 50 μm

Immunostaining of αSMA and PDGFRβ, common markers of activated fibroblasts and pericytes, was used to quantify the relative amount of activated fibroblasts in the tumor stroma. Although integrin α11 partially co-localized with αSMA in xenograft tumors in WT mice (Fig. 8c), no significant differences in the amount of PDGFRβ or αSMA expression (Fig. 8a-b and Additional file 1: Figure S1) in tumors in α11-KO compared to WT mice were found.

### Integrin α11β1 does not affect uptake of ^3^H-5FU

**Fig. 9:**
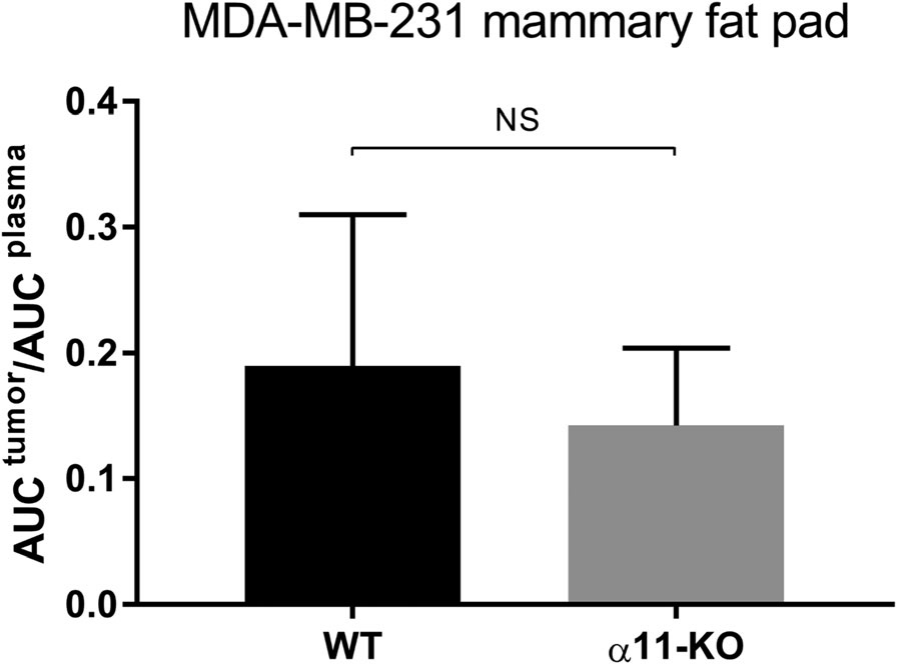
Microdialysis. Dialysate was sampled for 90 min after i.v. injection of ^3^H-5FU, and the ratio between ^3^H-5FU level in plasma and in MDA-MB-231 mammary fat pad tumor was calculated as Area Under Curve (AUC). There was no significant difference in uptake when WT and α11-KO mice were compared (*n* = 5). Mean ± SD

The reduced tumor PIF found in MDA-MB-231 mammary fat pad tumors in α11-KO mice was not associated with increased uptake of ^3^H-5FU measured by microdialysis (Fig. 9).

### Pressure homeostasis and integrin α11β1 in heterospheroids

**Fig. 10:**
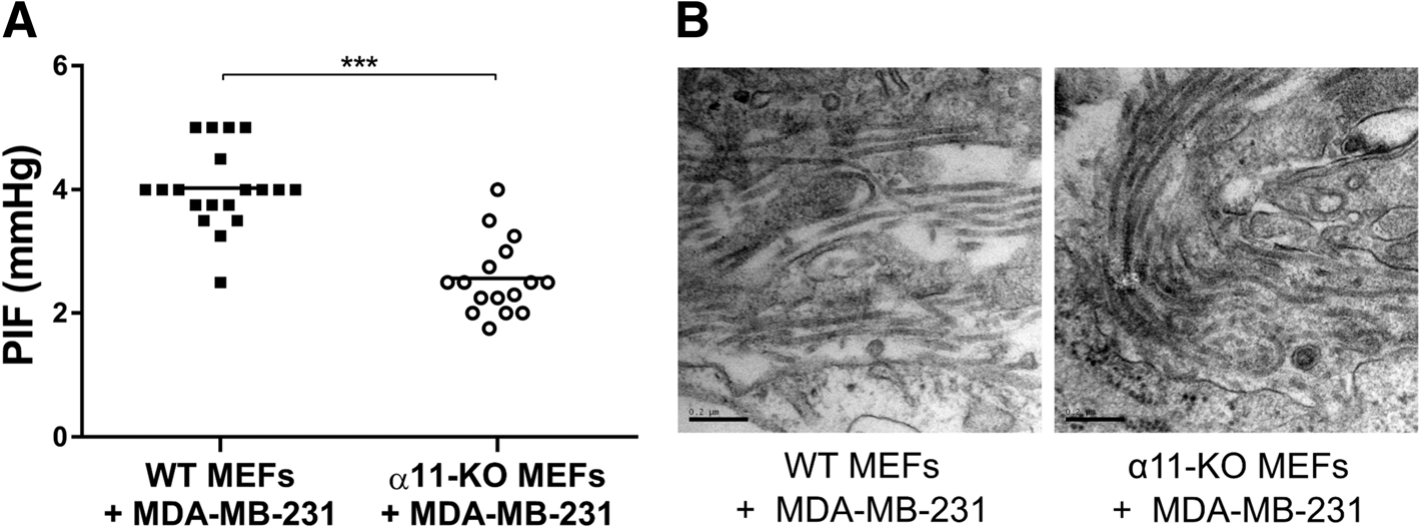
Heterospheroids. The individual interstitial fluid pressures (PIF) in heterospheroids containing a mixture of mouse embryonic fibroblasts (MEFs) and MDA-MB-231 breast tumor cells (4:1) (**a**). α11-KO MEFs + MDA-MB-231 spheroids showed a significant reduction in PIF compared to WT MEFs + MDA-MB-231 spheroids. *** *p* < 0.001. Transmission electron microscopy images show collagen fibrils in both heterospheroid types (**b**)

Since the in vivo results demonstrate that stromal integrin α11β1 has a role in maintaining pressure homeostasis in triple-negative breast xenograft tumors, we also investigated the isolated effect of integrin α11-positive fibroblasts on tumor PIF in a simplified system. Spheroids composed of fibroblasts lacking integrin α11 grown together with MDA-MB-231 cells had significantly lower PIF compared to spheroids with MDA-MB-231 cells and WT fibroblasts (Fig. 10a-b). These data indicate that the difference in PIF is, at least in part, due to integrin α11-positive fibroblasts.

### Tumor metastases

No lung metastases were seen when investigating coronal HE stained sections from lungs at end stage.

## Discussion

Integrins are essential adhesion receptors necessary for intercellular communication, attachment of cells to the ECM and modulation of the tumor microenvironment [36–39]. In this study, we have demonstrated that stromal integrin α11-deficiency markedly decreased PIF in vivo using two orthotopic human triple-negative breast cancer cell lines. A perturbed collagen structure was seen, with fewer aligned and thinner collagen fibrils. Furthermore, integrin α11-deficiency impeded orthotopic breast tumor growth in the MDA-MB-231 model, and the same trend was also found in the MDA-MB-468 orthotopic model. By investigating the isolated effect of integrin α11-positive fibroblasts on MDA-MB-231 tumor cells in vitro, we provide here evidence that PIF regulation is, at least partly, mediated by integrin α11-positive fibroblasts.

Integrin α11β1 has arisen as a possible marker of a pro-tumorigenic subset of CAFs in the tumor microenvironment [40, 41]. It has been found to be overexpressed in the stroma of lung cancer and head and neck cancer [40, 42]. Further, it stimulates lung cancer cell growth in vitro [20], and lung and prostate cancer growth in vivo [28, 33]. However, its role in tumor growth and progression is still not clear, especially in breast tumors where we recently reported that it did not affect the growth of the murine TNBC cell line 4 T1 in vivo [33].

In the present study, we found that stromal integrin α11-deficiency led to reduced tumor PIF in both orthotopic xenograft models. This demonstrates for the first time that integrin α11β1 has a role in maintaining an elevated PIF in solid tumors. A dense ECM, contractile fibroblasts, leaky blood vessels and dysfunctional lymphatic drainage are possible causes of increased PIF in tumors [8]. PIF can be actively modulated through interactions between contractile fibroblasts and ECM molecules [8, 23], where fibroblasts have been proposed to normally exert a tension on the collagen network through collagen-binding integrins [14]. Furthermore, integrin α11β1 contracts collagen matrices experimentally [17], and we therefore suggest that integrin α11β1-mediated PIF modifications can involve a contraction of the interstitial space mediated by direct or indirect binding of integrin α11-positive fibroblasts to collagen.

The involvement of integrin α11-positive fibroblasts in tumor PIF homeostasis is supported by our study of heterospheroids, where we observed a similar PIF reduction in spheroids composed of MDA-MB-231 cells and integrin α11-deficient fibroblasts. This simplified system allows us to investigate how fibroblasts grown together with tumor cells can influence PIF [18–20]. In line with our results, a similar integrin α11β1 function in pressure regulation has previously been shown in fibroblasts/lung cancer heterospheroids [20]. However, although these avascular spheroid studies indicate that the pressure regulatory abilities of integrin α11β1 is, at least in part, mediated by integrin α11-positive fibroblasts, the mechanisms behind integrin α11-mediated effect on PIF in heterospheroids are not investigated in detail in this study. In addition, we cannot exclude additional factors in the more complex in vivo system, such as influence of the tumor vasculature, which has been shown to have an important impact on tumor PIF [13, 43–45].

Furthermore, integrin α11-deficiency led to less organized and thinner collagen fibrils in the orthotopic models, which could be a contributing factor to reduced tumor PIF. Although it has been shown that the collagen-binding proteoglycan fibromodulin promotes the formation of a dense stroma and increased tumor PIF [46], it is nevertheless difficult to predict how different components in the extracellular matrix affect the hydraulic conductivity of tissues and thereby fluid flow and PIF [47].

Although the present study is the first to identify integrin α11β1 as participating in regulation of pressure in solid tumors, it is already known to maintain a homeostatic PIF in dermis [29, 30]. Furthermore, we have previously demonstrated the function of β1-integrins in the regulation of dermal PIF by inhibiting β1-integrins [21].

Numerous studies have highlighted the role of CAFs in tumor progression, invasion and metastasis, either directly by stimulation of tumor cells via production of pro-tumorigenic growth factors or indirectly by for example remodeling the ECM (reviewed in [48]). Here we show that integrin α11β1, known to be expressed during myofibroblast differentiation [25, 26], seems to facilitate breast tumor growth in vivo.

In previous studies, the pro-tumorigenic abilities of integrin α11β1 have been associated with increased matrix stiffness, collagen reorganization and increased levels of IGF-2 [28, 40]. In the present study, changes in pressure homeostasis and collagen organization could both influence tumor growth and invasion. Of interest, increased tumor PIF has been linked to tumor aggressiveness in some human cancers [9, 49], and is an independent poor prognostic factor in cervical cancer [10, 50].

There have been reports suggesting that increased tumor PIF can both facilitate and inhibit tumor progression. First, major pressure gradients due to increased tumor PIF can enhance interstitial fluid flow at and lymph drainage from the tumor margins, which probably increase the risk of cancer cells leaving the tumor. Increased flow can also facilitate tumor progression indirectly by either mechano-modulation of the tumor stroma or by changing the host immune response and thereby promote immunological tolerance (reviewed in [51, 52]). Further, in vitro elevation of tumor PIF increased proliferation of human osteosarcoma [53] and oral squamous cell carcinoma cells [54]. Similarly, in vivo lowering of tumor PIF, and thereby reduction of mechanical stretch for 24 h, reduced tumor cell proliferation in vulva and lung xenograft tumors [55]. However, contrary to these findings, increased tumor PIF may also limit uptake of nutrition and growth factors into the tumor and thereby inhibit tumor cell progression [8]. In the context of breast cancer, MDA-MB-231 cells have actually been shown to invade towards regions of higher pressure in vitro [56, 57], indicating that the elevated tumor PIF may in fact restrain breast tumor invasion. In summary, these findings demonstrate that maintenance of a high tumor PIF may be a contributing factor to integrin α11β1’s pro-tumorigenic effects, but at the same time, it can have opposite effects during tumorigenesis, pinpointing the need for further preclinical investigation.

Although increased tumor PIF can be a major barrier in cancer treatment, lowering of tumor PIF by integrin α11-deficiency did not increase the uptake of the low molecular weight drug ^3^H-5FU into MDA-MB-231 tumor interstitium. Low molecular weight compounds are transported by both diffusion and bulk flow/convection, and we have previously shown that reducing PIF can increase the uptake of the small molecular weight drugs ^3^H-5FU [11, 58] and ^51^Cr-EDTA [12, 59] into the tumor interstitium. However, in parallel with the results in the present study, it is evident that lowering of PIF will not always increase the uptake of low molecular weight drugs [35, 60]. Similarily, Flessner et al. showed that decapsulation of ovarian xenografts markedly decreased PIF to zero, but did not increase penetration of the high molecular weight drug trastuzumab into the tumor [61]. In summary, probably other features of the tumor microenvironment can also contribute to the failure of transport within solid tumors [5, 61].

Our data show that integrin α11-deficiency leads to thinner and less organized collagen fibrils in the orthotopic tumor stroma. Changes in collagen composition and organization are already known to influence tumorigenesis and can predict breast cancer behavior [3]. For example, progressive deposition of collagen [62] and increased collagen fiber linearization [63, 64] are associated with breast cancer aggressiveness.

While integrin α11-deficiency influenced tumor growth and reduced PIF with concomitantly more disorganized collagen fibrils in the orthotopic tumors, no effect was seen in the MDA-MB-231 ectopic tumors. Interestingly, there was similar amount of integrin α11β1 expression in both the MDA-MB-231 models. In a previous study, we also observed that while integrin α11-deficiency reduced RM11 tumor growth, but did not affect 4 T1 tumor growth, the integrin α11β1 expression was not higher in RM11 than in 4 T1 tumors [33]. Thus, differences in integrin α11β1-expression cannot explain the contrasting effect seen in these in vivo models.

The different effects seen between the MDA-MB-231 orthotopic and ectopic tumors show that tumor location significantly influences the effect of integrin α11β1 in vivo. The tumor microenvironment displays a significant heterogeneity [65], and the subcutaneous location probably does not always give rise to a representative tissue-specific stromal infiltration [66–68]. Supporting the fact that the organ-specific fibroblasts influence breast tumor growth differently, co-injection of breast fibroblast with breast tumor cells increased tumor growth, whereas no enhancement was seen with the co-injection of skin fibroblasts [69]. The significance of the local microenvironment illustrates the complexity of in vivo studies, and may explain some of the discrepancies seen with different mouse models. This underlines the importance of choosing the appropriate preclinical model, particularly when investigating the tumor microenvironment. If possible, orthotopic models should be preferred rather than ectopic ones.

## Conclusion

Our findings indicate an important role for integrin α11β1 in interstitial fluid pressure regulation in the breast tumor microenvironment. Further, since integrin α11β1 seems to impede breast cancer growth, it may be an interesting candidate for stromal targeted therapy.

## Additional file

Additional file 1: **Figure S1.** Collagen and activated fibroblasts in MDA-MB-231 subcutaneous tumors. The total fraction of Picrosirius-red, αSMA and PDGFRβ positive staining demonstrated no differences between MDA-MB-231 subcutaneous tumors in WT and α11-KO mice (*n* = 3 WT and *n* = 4 α11-KO). Mean ± SD. (TIF 242 kb)

## Abbreviations

^3^H-5FU: ^3^H-5-Fluorouracil
5FU: 5-Fluorouracil
AUC: Area under the curve
CAFS: Cancer associated fibroblasts
DAB: 3,3′-Diaminobenzidine
ECM: Extracellular matrix
HE: Hematoxylin and eosin
HER-2: Human epidermal growth factor receptor 2
IGF-2: Insulin-like growth factor 2
MAB: Monoclonal antibody
MBq: Megabecquerel
MEFs: Mouse embryonic fibroblasts
PAB: Polyclonal antibody
PBS: Phosphate buffered saline
PDGFRβ: Platelet-derived growth factor receptor beta
PIF: Interstitial fluid pressure
S.C.: Subcutaneous
SCID: Severe combined immunodeficiency
SEM: Scanning electron microscopy
TEM: Transmission electron microscopy
TNBC: Triple-negative breast cancer
WT: Wild type
α11-KO: Integrin α11-deficient
αSMA: Alpha-smooth muscle actin
