# Supplementary figures and images for: Stromal integrin α11-deficiency reduces interstitial fluid pressure and perturbs collagen structure in triple-negative breast xenograft tumors
Source: BMC Cancer. 2019 Mar 15;19:234. doi: 10.1186/s12885-019-5449-z (PMC6419843; doi:10.1186/s12885-019-5449-z)

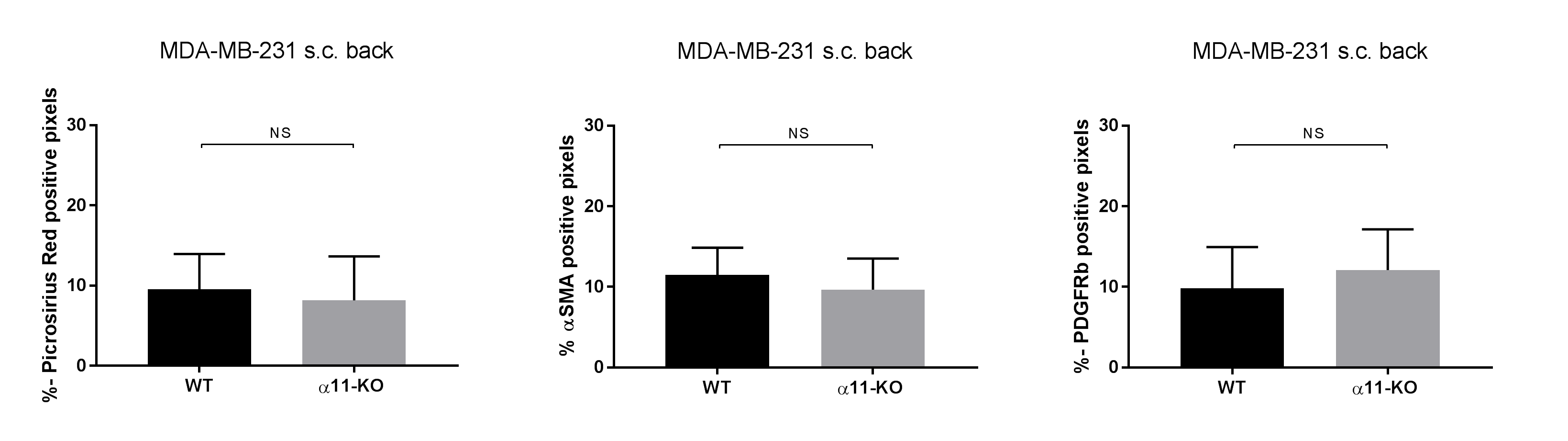

Supplement: Supplementary file 1 — Figure S1. Collagen and activated fibroblasts in MDA-MB-231 subcutaneous tumors. The total fraction of Picrosirius-red, αSMA and PDGFRβ positive staining demonstrated no differences between MDA-MB-231 subcutaneous tumors in WT and α11-KO mice (n = 3 WT and n = 4 α11-KO). Mean ± SD. (TIF 242 kb) [file 12885_2019_5449_MOESM1_ESM.tif]
